# Intoxication aiguë sévère par les pesticides organophosphorés: à propos de 28 cas

**Published:** 2011-03-01

**Authors:** Ali Derkaoui, Abderrahim Elbouazzaoui, Noufel Elhouari, Sanae Achour, Smael Labib, Hicham Sbai, Mustapha Harrandou, Mohammed Khatouf, Nabil Kanjaa

**Affiliations:** 1 CHU Hassan II Service d’Anesthésie - Réanimation, Fès, Maroc; 2 CHU Hassan II Laboratoire de Toxicologie Médicale et d’Analyse Toxicologique, Fès, Faculté des Sciences et Techniques, Kenitra, Maroc

**Keywords:** Intoxication, pesticides organophosphorés

## Abstract

**Abstract:**

Les pesticides organophosphorés (POP) sont des pesticides organiques de synthèse, largement utilisés en agriculture essentiellement comme insecticide, nemacide ou acaricide. Ce sont les produits agricoles, les plus incriminés dans les intoxications dans notre contexte. L’objectif de ce travail était de déterminer les caractéristiques cliniques, paracliniques, et évolutives de cette intoxication en milieu de réanimation. Étude rétrospective portant sur les cas admis en réanimation (2003-2010). Les critères d’inclusion étaient d’ordre clinique, para clinique, thérapeutique et évolutif. 28 cas ont été recensés: 19 femmes et 9 hommes, âge moyen= 24,5±11 ans. La tentative de suicide était le principal motif d’intoxication (19cas). Le Glasgow coma score était en moyenne de 11±4. Le syndrome central, était présent chez 78 % de nos patients, suivi du syndrome muscarinique 71% et le syndrome nicotinique dans 53% des cas. La prise en charge thérapeutique a consisté à la ventilation mécanique dans 50% des cas, l’usage des drogues vasoactives dans 14% des cas et l’administration d’un traitement antidotique dans 64 % des cas. La mortalité globale était de 25%. Les pesticides organophosphorés sont les toxiques agricoles, le plus souvent incriminés dans notre contexte. Les symptômes résultent d’une importante accumulation d’Acétylcholine (Ach) dans l’organisme; responsable de l’apparition des trois syndromes caractéristiques. Le diagnostic biologique se fait par le dosage de l’activité cholinestérasique dans le plasma. Le traitement associe des mesures symptomatiques qui reposent essentiellement sur la réanimation respiratoire et neurologique au traitement antidotique. L’évolution clinique dans ce type d’intoxication, est généralement favorable sous traitement avec régression des signes en quelques jours. Le décès est essentiellement, le fait d’une insuffisance respiratoire de mécanismes multiples: encombrement bronchique réalisant une véritable “noyade interne”, bronchoconstriction, paralysie des muscles respiratoires, œdème pulmonaire d’évolution gravissime.

## Introduction

Les pesticides organophosphorés (POP) sont des pesticides organiques de synthèse utilisés essentiellement comme insecticides. Cette classe chimique s’est considérablement développée durant la deuxième guerre mondiale avec la synthése du parathion et du malathion. Du fait de leur rémanence, les (POP) ont remplacé progressivement les organochlorés, très persistants dans l’environnement et très toxiques pour l’Homme. Toutefois l’utilisation des POP n’est pas sans risques comme le montre le nombre croissant de cas d’intoxication parfois gravissimes voire mortelles. Au Maroc, les données épidémiologiques établies par le centre anti-poison (CAPM) montrent que les (POP) sont responsables de 13 % d’intoxications tous toxique confondu [1]. Ce travail est une étude rétrospective ayant concerné tous les dossiers de patients admis au service de réanimation au CHU Hassan II de Fès pour intoxication au (POP) durant une période de 7 ans. Le but de notre travail était de décrire les caractéristiques cliniques, paracliniques et évolutives de nos patients et de les comparer avec les données de la littérature.

## Méthodes

C’est une étude rétrospective effectuée sur une période de 7 ans du mois de janvier 2003 au mois de Décembre 2010 et incluant 28 patients pris en charge dans le service de réanimation polyvalente du CHU Hassan II de Fès pour intoxication aux pesticides organophosphorés. Les paramètres épidémiologiques et les données cliniques, paracliniques, thérapeutiques et évolutifs de l’intoxication ont été analysés pour chaque cas. Les résultats ont été exprimés en pourcentage ou en moyenne ± écart-type.

## Résultats

**Figure 1:**
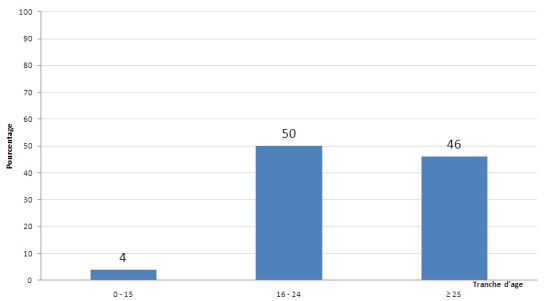
Répartitions des intoxications aux pesticides organophosporés chez un groupe de 28 patients pris en charge au CHU Hasssan II de Fès de 2003 a 2010 en fontion a l’âge

Nous avons recensé 28 patients durant la période concernée par l’étude s’étalant sur 7 ans, L’âge moyen des patients était de 24,5±11 ans, avec des extrêmes allant de 12 ans à 62 ans. La tranche d’âge la plus touchée est comprise entre 16 et 24 ans, soit 50 % (Figure 1).

Le sexe féminin était largement prédominant soit 67 % (sex-ratio de 2,11). L’intoxication était dans un but suicidaire chez 67 % des cas. Les pesticides organophosphorés sont les toxiques agricoles, le plus souvent incriminés dans notre étude.

La symptomatologie était déterminée par la survenue des trois syndromes caractéristiques: le syndrome central fait de (confusion, anxiété, convulsion et coma profond) était présent chez 78 % des patients. Le syndrome muscarinique fait de (myosis, une augmentation des secrétions bronchiques et bronchoconstriction et une augmentation des sécrétions salivaires, vomissement, diarrhée, et défécation involontaire et une bradycardie) a été retrouvé chez 71 % des cas. Le syndrome nicotinique fait essentiellement de fasciculation musculaire a été noté chez 5,3% des cas. Des troubles du rythme, étaient retrouvés chez 8,6% de nos patients, et un état de collapsus cardio-vasculaire chez 13 % des cas. Un patient dans notre série a présenté un syndrome intermédiaire.

Le dosage de l’activité anti-cholinéstérasique n’a pas été réalisé chez nos patients car pas disponible au CHU au cours de la période étudiée.

Le traitement symptomatique a été effectué chez tous nos patients. Le recours à l’intubation et ventilation artificielle (IVA) était nécessaire chez 50 % des cas. Tous nos patients ont bénéficié de remplissage vasculaire. Le soluté de remplissage vasculaire utilisé était le sérum salé à 0,9%. L’administration de drogues inotropes et/ou vasoactives, (dopamine, dobutamine, adrénaline), était indispensable chez 14 % des patients. Le traitement anticonvulsivant (Diazépam + phénobarbital), était administré chez 10% de nos patients. Le traitement évacuateur a consisté en un lavage gastrique chez 89% de nos patients. Le traitement antidotique à base d’atropine et/ou contrathion était administré dans 64 % des cas. La durée moyenne de l’hospitalisation variait de 3 à 8 jours.

Le taux de létalité était lourd (25 %), les facteurs de mortalité retrouvé dans notre étude sont: l’administration de drogues inotropes et ou vasoactive à l’admission (p=0,004) et la survenu d’une instabilité hémodynamique au cours de l’hospitalisation en réanimation (p=0,018) (Tableau 1).

## Discussion

Les pesticides organophosphorés sont les toxiques agricoles, le plus souvent incriminés dans notre étude. Ils sont responsables de la majorité des intoxications aigues dans les pays d’Asie [2].

Ce sont des pesticides organiques de synthèse largement utilisés en agriculture essentiellement comme: (insecticide, nemacide ou acaricide), leur mode d’action repose sur une inhibition des cholinestérases des insectes cibles, et ceci constitue également leur mode d’action toxique principale chez l’homme [3].

Cliniquement [2-4], les symptômes résultent d’une importante accumulation d’Acétyle –choline (Ach) dans l’organisme; responsable de l’apparition des trois syndromes caractéristiques, quelques minutes à quelques heures, après le début de l'exposition. Le syndrôme muscarinique: résulte d’une potentialisation de l’activité parasympathique post ganglionnaire sur les muscles lisses, le cœur et certaines glandes exocrines. Les signes dépendant de cet effet sont : un myosis, un syndrôme respiratoire, (constriction thoracique, augmentation des secrétions bronchiques et bronchoconstriction et une augmentation des sécrétions salivaires). Un syndrome digestif (nausée, vomissement, crampe abdominale, diarrhée, ténesme et défécation involontaire) et une bradycardie, qui peut évoluer vers un bloc auriculo-ventriculaire. Le syndrôme nicotinique: résulte de l’accumulation de Ach au niveau de la plaque motrice, et des synapses préganglionnaires du système sympathique, se manifeste par: une faiblesse musculaire pouvant affecter les muscles respiratoires et aggraver la difficulté respiratoire, une fasciculation musculaire, une tachycardie, qui peut masquer la bradycardie d’origine muscarinique et en fin par une hypertension artérielle. Le syndrôme central : qui se manifeste par un état confusionnel, anxiété, irritabilité, ataxie et parfois un coma convulsif. Dans notre série le syndrôme central, était présent chez 78 % de nos patients, suivi du syndrôme muscarinique 71 % et enfin le syndrome nicotinique dans 53 % des cas.

Quelques atteintes particulières, méritent d’être connues: Le syndrôme intermédiaire, récemment décrit, pouvant survenir 1 à 4 jours après la phase aiguë, caractérisé par un déficit moteur intéressant des territoires particuliers (muscles proximaux, muscles fléchisseurs du cou, les paires crâniennes et les muscles respiratoires). L’aspect particulier du syndrome intermédiaire, réside dans la gravité de l’atteinte respiratoire, rendant nécessaire la surveillance prolongée d’au moins quatre jours, de toute intoxication organophosphorée [5-7]. Dans notre série, ce syndrôme a été retrouvé chez un patient qui a présenté une réagravation respiratoire, 5 jours après son hospitalisation, ayant nécessité le recours à la ventilation mécanique, et chez qui l’évolution était favorable. La lésion cardiaque [2]: Se traduisant par la survenue progressive d’un état choc cardiogénique ou d’un bloc atrioventriculaire avec possibilité de survenue brutale d'une asystolie. Des troubles du rythme, étaient retrouvés chez 8,6 % de nos patients, et un état de collapsus cardio vasculaire chez 13 % des cas ayant une intoxication au POP, ce qui a fait toute la gravité de ce type d’intoxication dans notre série. Les Lésions pancréatiques [8]: Ont été également décrites lors d'intoxications aiguës graves aux POP. Elles mettent rarement en jeu le pronostic vital. Elles seraient relativement fréquentes et méritent d'être recherchées systématiquement.

Le diagnostic biologique se fait par le dosage de l’activité cholinestérasique dans le plasma, (test sensible et spécifique), qui revêt une importance capitale pour confirmer le diagnostic et déterminer la gravitée de l’intoxication [2,4]. Ce dosage n’était pas disponible pendant la période de l’étude.

Le traitement des intoxications organophosphorées associe des mesures symptomatiques au traitement antidotique. Le traitement symptomatique, repose essentiellement sur la réanimation respiratoire et neurologique. La décontamination est soit digestive, par un lavage gastrique même au delà de 2 heures en cas d’ingestion massive; soit cutanée, par un lavage à l’eau et au savon lors d’une exposition externe [2,4,9]. Le traitement repose sur les parasympatholytiques (Atropine) utilisés par voie intraveineuse directe après une bonne oxygénation afin d’éviter la survenue de fibrillation ventriculaire sur un cœur anoxique. La dose à administrer est fonction de la gravité de l’intoxication, du poids et de la réaction du patient. A titre indicatif : prescrire 2 à 4 mg par voie IV toutes les 10-15 minutes ou 0,015-0,05 mg/kg (chez l’enfant) jusqu'à apparition des signes d’atropinisation (bouche sèche, rougeur, tachycardie et mydriase) et maintenir une dose d’entretien de 0,02 mg/Kg/heure pendant 24 heures vu que l’atropine n’agit pas sur l’inhibition des cholinestérases. La pralidoxime (contrathion): Constitue le véritable traitement antidotique, un flacon contient 200 mg de pralidoxime à administrer le plus précocement possible, par voie intra-veineuse préférentielle, éventuellement sous-cutanée ou intramusculaire. On donne une dose de charge de 400 mg à 2 g en perfusion intraveineuse de 30 minutes (enfant : de 20 à 40 mg/kg), puis une dose d’entretien de 8 à 10 mg/kg/h. La durée du traitement est de 4 à 6 jours jusqu’ à 3 semaines dans certains cas. Des effets secondaires peuvent apparaître à type de troubles visuels : (diplopie, vision floue), malaise, vertiges, céphalées et en fin une tachycardie. Cette oxime permet une régénération des cholinestérases sanguines sans pour autant agir sur les cholinestérases cérébrales donc n’améliore pas les troubles de conscience lorsqu’il en existe [10-13].

L’évolution clinique dans ce type d’intoxication est généralement favorable sous traitement avec régression des signes en quelques jours. Le décès est essentiellement le fait d’une insuffisance respiratoire de mécanismes multiples: encombrement bronchique réalisant une véritable “noyade interne”, bronchoconstriction, paralysie des muscles respiratoires, œdème pulmonaire d’évolution gravissime [2,4,14]. Le traitement antidotique (Atropine) était administré chez 69,5% de nos patients. La mortalité dans notre série est de 25 %, elle varie de 4 à 10 % dans la littérature [15].

## Conclusion

L’intoxication à la POP est un véritable problème de santé au Maroc. Elle réalise une affection grave dominée par la détresse respiratoire et neurologique à l’origine de la plupart des décès. Elle concerne dans notre contexte surtout des femmes jeunes qui ingèrent le produit dans un but d’autolyse. Le diagnostic est basé sur la clinique et le dosage de l’activitée cholinestérasique dans le plasma. Le traitement associe des mesures symptomatiques qui reposent essentiellement sur la réanimation respiratoire et neurologique au traitement antidotique. L’évolution clinique dans ce type d’intoxication est généralement favorable sous traitement avec régression des signes en quelques jours. la mortalité est élevée dans notre contexte, de ce fait, elle doit être considérée comme une urgence diagnostic et thérapeutique. La disponibilité commerciale de ces produits devient inquiétante, justifiant le recours à un large programme de prévention pour informer le public et les autorités du danger des POP.

## Conflit d’intérêts 

Les auteurs ne déclarent aucun conflit d’intérêt.

## Contribution des auteurs 

Tous les auteurs ont contribué à la réalisation ce travail selon les critères de l’ICMJE.

## Figures and Tables

**Tableau 1:** Facteurs pronostiques de mortalité par l’intoxication aux pesticides organophosphorés aux chez un groupe de 28 patients pris en charge au CHU Hassan II de Fès de 2003 à 2010

**Paramètres**	**Survivants**	**Décédés**	**p**
instabilité hémodynamique	11%	89%	0,018
Détresse respiratoire	63,6%	36,4	0,7
Signes digestifs	50%	50%	0,12
Convulsion	66,7%	33,3%	0,14
Intubation ventilation artificielle	64,3%	35,7%	0,66
Drogues inotropes/et ou vasoactives	0%	100%	0,004

P< 0 ,05 est significatif
